# On the edge of the social media landscape: associations with adolescent substance use and moderation by parental rules

**DOI:** 10.1093/pubmed/fdae290

**Published:** 2024-11-19

**Authors:** Hanan Bozhar, Susanne R de Rooij, Anja Lok, Tanja Vrijkotte, Helle Larsen

**Affiliations:** Department of Public and Occupational Health, Amsterdam Public Health Research Institute, Amsterdam University Medical Centers, University of Amsterdam, Van der Boechorststraat 7, 1081 BT Amsterdam, The Netherlands; Centre for Urban Mental Health, Institute for Advanced Study, University of Amsterdam, Oude Turfmarkt 147, 1012 GC Amsterdam, The Netherlands; Centre for Urban Mental Health, Institute for Advanced Study, University of Amsterdam, Oude Turfmarkt 147, 1012 GC Amsterdam, The Netherlands; Department of Epidemiology and Data Science, Amsterdam University Medical Centers, University of Amsterdam, 1105 AZ Amsterdam, The Netherlands; Amsterdam Public Health research institute, Aging & Later life, Health Behaviors & Chronic Diseases, 1081 BT Amsterdam, The Netherlands; Amsterdam Reproduction and Development Research Institute, 1105 AZ Amsterdam, The Netherlands; Centre for Urban Mental Health, Institute for Advanced Study, University of Amsterdam, Oude Turfmarkt 147, 1012 GC Amsterdam, The Netherlands; Department of Psychiatry, Amsterdam University Medical Centers, University of Amsterdam, 1105 AZ Amsterdam, The Netherlands; Department of Public and Occupational Health, Amsterdam Public Health Research Institute, Amsterdam University Medical Centers, University of Amsterdam, Van der Boechorststraat 7, 1081 BT Amsterdam, The Netherlands; Centre for Urban Mental Health, Institute for Advanced Study, University of Amsterdam, Oude Turfmarkt 147, 1012 GC Amsterdam, The Netherlands; Amsterdam Reproduction and Development Research Institute, 1105 AZ Amsterdam, The Netherlands; Amsterdam Public Health Research Institute, 1081 BT Amsterdam, The Netherlands; Centre for Urban Mental Health, Institute for Advanced Study, University of Amsterdam, Oude Turfmarkt 147, 1012 GC Amsterdam, The Netherlands; Department of Psychology, University of Amsterdam, 1018 WS Amsterdam, The Netherlands

**Keywords:** adolescents, parental rules, social media, substance use

## Abstract

**Background:**

Adolescent problematic social media use (PSMU) has been increasing. Digital engagement has been associated with substance use, but little is known about the potential protective role of parents. We investigated whether screen and substance-related parental rules moderated the associations between (problematic) SMU and intake of tobacco, alcohol, hashish/marijuana, and laughing gas.

**Methods:**

We used data from the Amsterdam Born Children and Development study (*N* = 1787; *M*age = 15.86 years; *SD* = 0.36). Both frequent and problematic SMU in relation to tobacco, alcohol, hashish/marijuana, and laughing gas intake levels; and moderation by perceived parental rules (screen/substances), was tested with ordinal logistic regression models.

**Results:**

PSMU was associated with higher chances of higher substance use levels. Hashish/marijuana use and heavy drinking were less prevalent in adolescents reporting the presence of parental rules on alcohol/drugs, compared to adolescents reporting no rules. Although parental rules on alcohol/drugs, but not screen time, moderated the relationship between PSMU and both hashish/marijuana use and heavy drinking, the moderation effect was modest, especially in mitigating substance use at higher PSMU-scores.

**Conclusion:**

PSMU was positively associated with a wide range of substance use behaviours. The potential significant role of parental rules (alcohol/drugs) mitigating these associations are highlighted.

## Introduction

Worldwide, alcohol, cannabis, and tobacco/nicotine products are popular substances among adolescents. Although substance use onset decreased during the COVID-19 pandemic, heavy substance use increased and the high levels of adolescent substance intake have stagnated in the period 2021–2022 in the United States.^1^ In 2021, half of Dutch adolescents reported to have consumed alcohol on at least one occasion, and among those consuming alcohol in the past month, 75% had been drinking heavily.^2^ About a quarter of the 16-year-olds had experience with smoking and cannabis. There are concerns among Dutch paediatricians regarding the high alcohol and drug abuse in adolescents which, sometimes even lead to hospitalization.^3^

Making irrational decisions and risk-taking behaviour increase during adolescence.^4^^,^^5^ The influence of peers has a prominent place in experimenting with substances which may lead to continuation of this behaviour.^6^ Moreover, the social–emotional changes in the brain during adolescence often enhance reward-seeking behaviour.^4^ This may explain adolescents’ desire and sensitivity to imitate behaviour that is perceived as normative or valued by others, as previous neurological studies for instance have demonstrated that being socially accepted activates the reward system in the brain.^7^

Nowadays such peer norms are also shared on social media, which is acting as a powerful driver for maintaining social connectedness.^6^ The use of various newly emerged social media platforms (e.g. TikTok, Instagram, Snapchat) has increased immensely among adolescents.^8^ Adolescents spend on average three hours a day on social media and use about three or more different social network sites.^9^ Social media provides adolescents with an anonymous environment for exploration of risky behaviour.^10^ This has raised concerns about the role of social media in the positive portrayal of substance use by online peers for instance.^11^^,^^12^ We need more insight in potential protective factors for adolescent risky substance use, as the availability of various substances and rapid evolvement of the online landscape continues.^13^

The frequency of social media use (SMU) has been positively linked to substance use.^6^^,^^13^^,^^14^ However, to the best of our knowledge, no studies have investigated the relationship between problematic SMU (PSMU) and the use of a broad spectrum of substances. Most studies investigated the relationship between SMU-frequency and for instance alcohol consumption only^15^^,^^16^ or focused on digital media use per se^6^^,^^17^^,^^18^ and not its problematic form, as assessed with the Social Media Disorder Scale.^19^ Social media use can be beneficial for development and social interactions,^20^ however when social media use becomes problematic it can be related to anxiety and depressive symptoms.^21^ PSMU resembles ‘addiction like’ symptoms as adolescents’ experience of pre-occupation and compulsive behaviour (i.e. difficulties with regulating social media use).^20^^,^^22^^,^^23^

Studies on the potential protective role of parental rules regarding both substances and especially digital media use on substance use behaviour among adolescents are scarce. Strict parental rules on alcohol-use have been found to be effective in minimizing use of multiple substances.^24^ Setting clear boundaries might prevent adolescents from approaching specific social hotspots in which substance use is common, mitigating the exposure to positive social norms related to substance use.^25^ Research on Internet-specific rules in preventing deviant online behaviour, such as timing and application restrictions, are limited and results are inconclusive.^26^ For instance, strict parental rules about internet did not appear to prevent sleep problems due to digital media for adolescents who were highly engaged in SMU.^27^ Screen-related rules could help limit overall exposure to online environments where substance use is normalized, thereby potentially reducing the likelihood of adolescents engaging in similar behaviours.^28^ Screen-related parental rules may not only limit overall media exposure but also reflect broader parental monitoring and control, which may shape how adolescents engage with social media content. Hence, we apply a broader approach, including both screen and substance-related rules. We hereby recognize that SMU and substance use behaviours might be interconnected and consequently their relationship may be impacted by both types of parental rules.

Therefore, the primary objective was to investigate whether screen and substance-related parental rules moderated the associations between SMU-frequency as well as PSMU and intake levels of tobacco, alcohol, hashish/marijuana, and laughing gas use. We explored whether the presence of either of these types of parental rules decreases the positive relationships between (problematic) SMU and intensity of substance use in adolescents (See online supplementary material for a colour version of this figure, Fig. S1). We controlled for demographic factors, peer problems, and parental substance.

## Methods

### Study population

We used data from the Amsterdam Born Children and their Development Study (ABCD).^29^ The ABCD study is a large-scale longitudinal study, which started in 2003 when 12 373 pregnant women living in Amsterdam were invited during prenatal consultation to complete a questionnaire (See online supplementary material for a colour version of this figure, Fig. S2). About 8000 children born in Amsterdam enrolled in this cohort-study among which physical and mental health were examined over time. During Phase-5 (2019–2020), in total 2291 adolescents aged 15–16 years and their parents (2189 mothers/1418 fathers) participated. Participants completed a digital questionnaire covering multiple topics including lifestyle, substance use behaviours, and digital media. We excluded participants (*N* = 504) with missing values on frequency of SMU and PSMU. Values on all other measures were complete. We performed analyses on a final sample of *N* = 1787.

### Measures

#### Social media use

The *frequency of SMU* was assessed asking adolescents about their daily use (viewing, responding, sharing/posting) of social network sites (*0 = never to 6 = 40+ times*). *Problematic SMU* was measured on the 9-item Social Media Disorder-Scale (SMDS) to indicate the level of addiction to social media with a yes/no response on each item (i.e. *conflict with family due to SMU*; *escape negative feelings through SMU*).^19^^,^^30^ We used the total sum score of all items (range = 0–9). Given the dichotomous nature of the data, we used polychoric correlation matrix to calculate ordinal alpha’s for reliability.^31^ The internal consistency for the SMDS-scale was good (ordinal α = 0.81). To visualize PSMU in figures, we used a recoded SMDS-score containing three categories (0–1; 2–3; 4–9) due to small frequencies of the higher scores.

#### Substance use behaviours

*Tobacco use* was assessed by asking participants how often they had ever smoked cigarettes/cut tobacco and water pipe. *Heavy alcohol use* was measured by asking how often they had consumed five or more units of alcohol on one occasion in the last month. Additionally, participants were asked how often they had ever used *hashish/marijuana* and *laughing gas*. Quantity of substance use was indicated based on the number of consumed alcohol units and smoked cigarettes per week. We considered the lowest categories, indicating ‘less than one unit or one time of usage’, as none use (Table 1).

**Table 1:** Descriptions of the utilization of social media and substances, and adolescents’ perceptions of rules (N = 1787)

Variable	N (%)	Mean (SD)
SMU/day **≥**1 time	1615 (90.4)	
Problematic SMU (0-9)		1.33 (1.44)
Cigarette smoking **≥**1 time	364 (20.4)	
Smoking/week **≥**1 cigarette	169 (9.5)	
Water pipe smoking **≥**1 time	179 (10.0)	
Alcohol units/week **≥**1 unit	569 (31.8)	
Heavy alcohol use **≥**1 time	379 (21.2)	
Hashish/marijuana use **≥**1 time	547 (30.6)	
Laughing gas use **≥** time	133 (7.4)	
Smoking allowed by parents		
*No*	1379 (77.2)	
*Yes or don’t know*	408 (22.8)	
Alcohol allowed by parents		
*No*	887 (49.6)	
*Yes or don’t know*	900 (50.4)	
Drugs allowed by parents		
*No*	1522 (85.2)	
*Yes or don’t know*	265 (14.8)	
Screen time rules present		
*Yes*	413 (23.1)	
*No*	1374 (76.9)	

*^Note^* ^: Alcohol use was reported over the prior month. SMU = social media use.^

#### Parental rules

We assessed perceived rules by asking adolescents: *Do your parents allow you to smoke?* (yes/no/I don’t know); *Do your parents allow you to drink alcohol?* (yes/no/I don’t know); *Do your parents allow you to use drugs?* (yes/no/I don’t know). We dichotomized the responses on these three items to reflect the perceived strictness of parental oversight (1= ‘not allowed by parents’ and 0= ‘allowed by parents’ or ‘I don’t know’). The inclusion of ‘I don’t know’ in the ‘allowed by parents’ category was based on the assumption that a lack of clear rules or communication from parents is functionally equivalent to allowing the behaviour. Additionally, adolescents were asked whether their parents have rules about how many hours a day they can watch TV, play (online) games, and use a laptop/tablet or mobile phone (1 = yes; 0 = no).

#### Peer problems

Peer problems were measured on a 5-item subscale of the Strengths and Difficulties Questionnaire (SDQ).^32^ We included the total sum score (range = 0–10; ordinal α = 0.72).

#### Socio-demographics

Socio-demographic factors were assessed and included participants’ sex (boy/girl), physiological ethnicity based on grandmother’s country of birth (1 = Dutch; 2 = Western other; 3 = Non-western), and secondary educational level (1 = Vocational; 2 = Senior general; 3 = Pre-university).

#### Parental factors

Parents were asked about the number of cigarettes smoked per day; the number of alcohol units consumed per week; and how often they used cannabis. We also included highest attained educational level (low/medium/high).

### Statistical analysis

To assess substance use behaviours of adolescents according to their SMU, we used multivariable ordinal logistic regression analysis conducted separately for: cigarette smoking, weekly cigarette smoking, and water pipe smoking (3 models); weekly alcohol consumption and heavy drinking (2 models); and ever use of hashish/marijuana and laughing gas use (2 models). All analyses were controlled for potential confounders: age, sex, educational level, ethnicity, and peer problems.

To test the moderating role of parental rules, we included interaction terms between both SMU-frequency and PSMU predictors, as well as screen and substance-related parental rules, as predictors for each substance separately (smoking/alcohol/drugs) using ordinal logistic regression, while controlling for all covariates and main effects (*P*-value ≤0.013 after Bonferroni correction). All analyses were performed with IBM SPSS Statistics 26.

## Results

### Demographics

Participants were on average 15.86 years of age (*SD* = 0.36; range = 15.14–16.87 years) of which 762 boys (42.6%) and 1025 girls (57.4%). Majority was native Dutch (*N* = 1346; 75.3%), while 12.5% and 12.2% had non-Western or other Western backgrounds, respectively. Pre-university attendance was highest (*N* = 1092; 61.1%), followed by senior-general (23.9%) and vocational levels (14.9%). Peer problem experience level was quite low within the sample (*M* = 1.32; *SD* = 1.40). Participants actively used social network sites on average 6-10 times a day. The average SMDS-score indicating PSMU was 1.33 (*SD* = 1.44). A SMDS-score of at least five indicates a problematic user.^19^ The proportion of problematic-users in the current sample was 3.4%. See Table 1 for further sample details. Most parents were highly educated and did not use cannabis. Mothers and fathers smoked, on average, less than one cigarette per day; and consumed 6 and 9 units of alcohol per week, respectively (Table S1).

### Substance use behaviours

Both more frequent and problematic SMU were associated with an increased likelihood of cigarette smoking, heavy drinking, hashish/marijuana use, and laughing gas use at higher frequency levels. Similarly, adolescents with higher SMU-frequency and PSMU were more likely to smoke a larger number of cigarettes and drink more alcohol units per week. Water pipe smoking at higher frequency levels was more likely among adolescents with higher PSMU (Table 2).

**Table 2:** Substance use by daily and problematic SMU adjusted for covariates

	Smoking behaviour			Alcohol consumption (last month)	Drug use		
	Cigarette smoking (times) OR (95% CI)	Smoking/week (X cigarettes) OR (95% CI)	Water pipe smoking (times) OR (95% CI)	Heavy alcohol use (times) OR (95% CI)	Alcohol/week (X units) OR (95% CI)	Hashish/marijuana use (times) OR (95% CI)	Laughing gas use (times) OR (95% CI)
SMU/day (times)	1.17^*^ (1.09-1.26)	1.22^*^ (1.10-1.35)	1.10 (1.00-1.21)	1.24^*^ (1.15-1.34)	1.21^*^ (1.14-1.29)	1.18^*^ (1.11-1.26)	1.15^*^ (1.03-1.29)
Problematic SMU	1.20^*^ (1.10-1.30)	1.16^*^ (1.04-1.29)	1.17^*^ (1.05-1.30)	1.11^*^ (1.02-1.20)	1.10^*^ (1.02-1.18)	1.10^*^ (1.02-1.19)	1.15^*^ (1.03-1.30)
Sex							
*Boys*	0.87 (0.68-1.12)	0.89 (0.63-1.25)	1.68^*^ (1.22-2.32)	0.82 (0.65-1.05)	0.77^*^ (0.63-0.95)	1.63^*^ (1.32-2.01)	1.34 (0.93-1.94)
*Girls (ref.)*							
Age	1.70^*^ (1.23-2.37)	2.32^*^ (1.47-3.65)	0.981 (0.64-1.51)	2.11^*^ (1.52-2.92)	2.24^*^ (1.69-2.98)	1.79^*^ (1.35-2.38)	2.16^*^ (1.31-3.55)
Education							
*Pre-university*	1.06 (0.71-1.58)	0.79 (0.47-1.32)	0.40^*^ (0.26-0.61)	1.19 (0.80-1.75)	0.98 (0.70-1.35)	1.34 (0.95-1.89)	0.67 (0.40-1.11)
*Sr. general*	1.51 (0.99-2.32)	1.31 (0.76-2.24)	0.70 (0.45-1.10)	1.11 (0.72-1.71)	1.04 (0.72-1.50)	1.75^*^ (1.20-2.54)	1.01 (0.59-1.72)
*Vocational (ref.)*							
Ethnicity							
*Non-Western*	0.25^*^ (0.15-0.44)	0.36^*^ (0.18-0.72)	1.06 (0.66-1.71)	0.24^*^ (0.14-0.43)	0.19^*^ (0.12-0.30)	0.27^*^ (0.17-0.43)	1.21 (0.72-2.05)
*Western other*	1.03 (0.73-1.47)	1.01 (0.62-1.64)	1.22 (0.76-1.95)	0.87 (0.61-1.24)	0.79 (0.58-1.08)	1.10 (0.81-1.49)	0.16 (0.68-1.98)
*Dutch (ref.)*							
Peer problems	0.82^*^ (0.74-0.90)	0.87^*^ (0.76-0.99)	0.90 (0.80-1.02)	0.78^*^ (0.70-0.86)	0.78^*^ (0.72-0.85)	0.85^*^ (0.78-0.92)	0.97 (0.85-1.10)

*Note:*  ^*^P < 0.05

^-^Frequency of daily SMU activities (0 = never; 1 = 1–2 times a day; 2 = 3–5 times a day; 3 = 6–10 times a day; 4 = 11–20 times a day; 5 = 21–40 times a day; 6 = more than 40 times a day).

^-^Frequency of smoking cigarettes/cut tobacco (0 = never; 1 = number of times per year; 2 = approximately once a month; 3 = number of times per month; 4 = once a week; 5 = number of times per week; 6 = daily).

^-^Number of cigarettes per week (0 = none; 1 = 1 to 4 per week; 2 = 5 to 7 per week (approximately 1 per day); 3 = 6 to 10 per week (about half a pack); 4 = 11 to 20 per week (about a pack); 5 = 21 to 40 per week (about 2 packages); 6 = 40 to 60 per week (about 3 packages); 7 = more than 60 per week (>3 packages)).

^-^Frequency of smoking water pipe (0 = never; 1 = 1–3 times; 2 = 4–10 times; 3 = 11–19 times; 4 = more than 19 times).

^-^Number of alcohol units per week during the last month (glasses, bottles or cans of alcohol): 0 = 0; 1 = 1–2; 2 = 3–5; 3 = 6–10; 4 = 11–15; 5 = 16–20; 6 = 21–30; 7 = more than 30.

^-^Frequency of heavy alcohol use during the last month (5+ units per occasion): 0 = not; 1 = 1–2 times; 2 = 3–5 times; 3 = 6–9 times; 4 = 10 times or more.

^-^Frequency of hashish/marijuana use: 0 = never; 1 = 1–3 times; 2 = 4–10 times; 3 = 11–19 times; 4 = more than 19 times.

^-^Frequency of laughing gas use (nitrous oxide): 0 = never; 1 = 1–3 times; 2 = 4–10 times; 3 = 11–19 times; 4 = more than 19 times.

To rule out the potential role of parental substance use and educational level, we conducted the main analyses controlling for these factors (tobacco/alcohol/cannabis separately) in addition to the other potential confounders and we found similar patterns of findings regarding the associations between adolescent (problematic) SMU and substance use behaviours.

### Moderation by parental rules

**Fig. 1:**
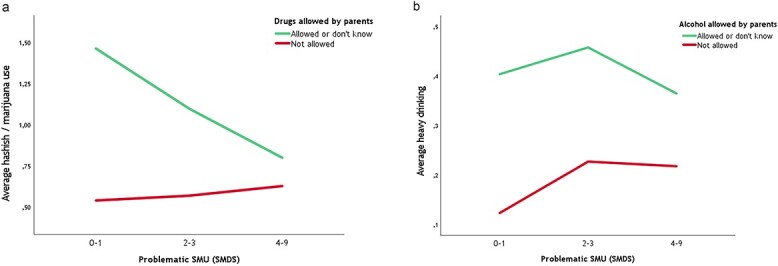
(a) Hashish/marijuana use by problematic SMU. (b) Heavy alcohol use by problematic SMU. Note: Visualization of (a) the average number of times participants used hashish/marijuana and (b) average number of times participants have engaged in heavy drinking in the prior month according to Problematic SMU and perceived rules regarding drugs and alcohol, respectively. Please see Table 3 for moderation statistics.

Adolescents reporting the presence of rules regarding drugs and alcohol showed overall less hashish/marijuana use and less heavy drinking compared to adolescents reporting no rules (Fig. 1a and b). The results showed small moderating effects of these rules on the relationship between PSMU and both hashish/marijuana use and heavy drinking (Table 3). Specifically, parental rules appeared not to mitigate hashish/marijuana use (B = 0.302; *P* = 0.002) and heavy alcohol use (B = 0.214; *P* = 0.010) in adolescents with higher PSMU-scores. The effect sizes indicate that while parental rules do moderate these associations, the strength of their impact is modest. Screen-related parental rules did not moderate any of the associations between SMU overall and substance use behaviours.

## Discussion

### Main findings of this study

The present study advanced our understanding of SMU in relation to adolescent use of various substances and the potential protective role of parental rules.

Overall, the findings suggest that both SMU-frequency as well as PSMU is positively associated with using various substances at higher intake levels. It appears that parental rules on drugs and alcohol may have a reduced likelihood of influence when adolescents experience PSMU-related behaviour. Notably, these findings should be interpreted with caution due to the small effect sizes. Nonetheless, our results suggest that hashish/marijuana use and heavy drinking remains lower overall when parental rules on substances are present. None of the other investigated interactions between (problematic) SMU and screen and substance-related parental rules were found to significantly moderate substance use behaviours.

### What is already known on this topic

Our study confirms prior findings linking increased digital engagement with higher alcohol use likelihood,^6^^,^^14^ and initiation of cannabis and tobacco intake.^17^ Online glorification and normalization of substance use might put adolescents at higher risk to engage in (long-term) substance use behaviours. Miller et al.^33^ underline the role of online peer groups and the increased risk for adolescents to progress towards more problematic habits of substance use offline. The dynamic evolving landscape of online interaction and the rise in popularity of various social platforms acts as the ideal market for substance supply and demand due to its free and anonymous nature.^34^ The absence or lack of regulations on social media makes advertisements/content glorifying substances easily accessible for a larger public, including minors.^35^ This may underscore the relevance of the parental role in adolescent online behaviour, awareness of associated risks, and clear rule-setting regarding alcohol and drug use.

The use of digital media plays an important role in adolescent identity-formation.^36^ Strict parental monitoring in this case may lead to adverse behaviour of adolescents trying to bypass their parents’ restrictions in order to stay socially active and autonomous.^37^ Sensitive parenting and attuned communication can protect adolescents against behavioural problems and deviant influence.^38^ What is considered normative by adolescents is likely to be identified by using peer online behaviour (posting/sharing content) as a valuable source of information to establish a behavioural pattern that aligns with the mainstream.^35^ Parents however can provide a different perspective on those social peer norms.

**Table 3:** Moderation by parental rules

*Smoking behaviour*	Cigarette smoking (times)		Smoking/week (X cigarettes)		Water pipe smoking (times)		
	*Beta*	*P-value*	*Beta*	*P-value*	*Beta*	*P-value*	
Times SMU/day x Rules screen time	−0.008	0.942	−0.068	0.658	0.108	0.395	
Times SMU/day x Rule smoking	0.035	0.680	0.088	0.419	0.181	0.091	
Problematic SMU x Rule screen time	0.008	0.929	−0.014	0.911	−0.063	0.578	
Problematic SMU x Rule smoking	−0.012	0.892	0.009	0.937	−0.022	0.841	
*Alcohol consumption (last month)*	Heavy alcohol use (times)		Alcohol/week (X units)				
	*Beta*	*P-value*	*Beta*	*P-value*			
Times SMU/day x Rule screen time	0.001	0.990	−0.008	0.928			
Times SMU/day x Rule alcohol	0.014	0.870	0.006	0.931			
Problematic SMU x Rule screen time	−0.047	0.630	−0.113	0.181			
Problematic SMU x Rule alcohol	0.214^*^	**0.010**	0.142	0.051			
*Drug use*	Hashish/marijuana use (times)		Laughing gas use (times)				
	*Beta*	*P-value*	*Beta*	*P-value*			
Times SMU/day x Rule screen time	0.003	0.971	−0.080	0.724			
Times SMU/day x Rule drugs	0.031	0.686	0.023	0.866			
Problematic SMU x Rule screen time	0.008	0.923	−0.433	0.108			
Problematic SMU x Rule drugs	0.302^*^	**0.002**	0.321	0.058			

*Note:*  ^*^P ≤ 0.013 after Bonferroni correction (0.05/4). Moderation analyses adjusted for: age, sex, educational level, ethnicity, and peer problems.

### What this study adds

Our findings extend prior research by demonstrating that PSMU predicts increased likelihood of multi-substance use at higher rates. Compared to SMU-frequency, PSMU captures adolescents’ experience of compulsive behaviour and personal feelings toward social media.

Parental substance rules moderated associations between PSMU and hashish/marijuana use and heavy drinking, aligning with prior research on alcohol-specific rules’ efficacy in mitigating adolescent substance use.^39^ However, we did not find moderation by screen-related parental rules. In contrast to rule-setting related to alcohol use, there is rather inconsistent and less evidence of internet-related rules, including screen time restrictions, mitigating adolescents’ PSMU.^20^ Social media-specific monitoring by parents was previously found to slightly moderate the association between SMU and alcohol use negatively.^40^ In the present study, we did not measure screen-related rules regarding the use of specific social media platforms or viewing certain content, but rather generally asked whether their parents imposed time restrictions regarding various digital practices (TV, gaming, laptop/tablet/phone use). This could explain the absence of a significant moderation effect of screen-related rules in the relationships between (problematic) SMU and substance use behaviours. Moreover, the ongoing debate on screen-related mental health issues and comorbid substance use behaviours highlights the need to look beyond mere screen exposure.^28^ It is essential to consider the complex process of identity formation that adolescents undergo in the digital age, where the integration of online and offline experiences can significantly influence behavioural patterns.^28^ Exploring both substance and screen-related rules as indicator of broader parental supervision remain important to understand the potential role of these rules.

Compared to the substance-related rules (whether using substances is allowed or not), the screen-related rule is less restrictive and specific by nature which might also impact the effectiveness depending on the parenting strategy that is applied^26^ (e.g. enforcement of rules). It is unclear to what extent screen time restrictions might protect against specific online content-exposure and substance use. If we hypothesize that (problematic) SMU influences offline substance use behaviours, we need to consider a potential time interval. It has been suggested that the benefit of parental rules is limited to the moment and context if they are simultaneously sanctioned.^40^ Hence, adolescents might not translate screen-related rules directly into a behavioural change regarding their substance intake.

While the moderating effect of screen-related rules was not significant in our study, including this variable offers important insights into the broader parental supervision framework, which may play a role in adolescents’ interaction with both online environments and substance use behaviours. By investigating this, we aim to provide a more comprehensive understanding of the complex role parental rules play in adolescent development.

When it comes to educating children about the risks of social media and substances, parental involvement may be essential. Weak or absent parental rule-setting has been found to be associated with increased substance use among adolescents.^41^ In the present study it appeared that parental rules on drugs and alcohol might be slightly less effective in mitigating hashish/marijuana use and heavy alcohol use among adolescents with higher PSMU. A longitudinal study suggests that intervening is mostly relevant prior to an already existing pattern of substance use and engagement with (co-) using friends, as one year after follow-up parental monitoring was no longer effective.^25^ Hence, parental rule-setting and monitoring might be particularly effective at an early stage when structural problem behaviour is still absent.

According to developmental theories, the environment in which adolescents thrive is proposed to reciprocally interact with their behaviour, such that a nurturing atmosphere in the social circle can enhance adolescents’ personal growth and resilience.^42^^,^^43^ Higher PSMU could indicate a stronger susceptibility to social approval and adherence to online peer behaviour (expected norms) resulting in parental rules regarding substances being less effective, and peer values taking primacy. Although the overall influence of parents on adolescent SMU is moderate, it is essential to set appropriate rules resonating with the adolescent life-stage in a context of positive parenting.^20^ Open parent–child communication, characterized by an affectionate and mindful relationship, has been found to be most effective in preventing PSMU overall.^20^

Strict parental rules on phone use, for instance, have been found to be only effective in adolescents with lower SMU.^27^ Additionally, adolescents strive for more autonomy in their decision-making which could explain the distancing from parental perspectives and rules.^44^ Parents themselves also tend to lower their supervision when their children reach late adolescence, which can make already vulnerable adolescents even more at risk for deviant influence.^44^ Hence, a sustainable approach of appropriate rule-setting prior to transitioning into a problematic state of both social media and substance use seems desirable.

### Limitations of this study

The present study has several limitations. The findings are not generalizable to broader geographic and demographic contexts, as our study sample predominantly consisted of Caucasian, higher educated adolescents from a particular region in the Netherlands. Additionally, the use of cross-sectional data restricts the ability to investigate longitudinal relationships and rule out causal or reciprocal effects.

The absence of content-based data makes it difficult to determine whether adolescents are exposed to substance-related content and whether it translates to offline behaviour as we only had self-reported measures available. Moreover, the prevalence of PSMU and intense substance use was low in our study population. Parental rules as measured in this study provided limited insight into the extent of strictness of, enforcement and adherence to these rules. This study only considered the presence of rather generic restriction rules. More detailed data was unfortunately not available. This also applies to the quantification of tobacco, alcohol, hashish/marijuana, and laughing gas use where adolescents had to choose between rather wide ranges of use divided over multiple categories. This could have limited the ability to get a more thorough indication of actual amount and recurrence of substance use, which may have limited the accuracy of the findings.

We recommend future research to further investigate the role of parental rules regarding screen and social media engagement by using more fine-grained data on parental rules as well as analysing longitudinal content-based data, including the patterns of use and what adolescents do and are exposed to on social network sites. Especially, the use of objectively measured data, indicating for instance actual engagement on specific social media platforms or interest for certain content, could enhance our understanding of adolescent online behaviour.

## Supplementary Material

Figure_S1_Research_model_23-10-2024_fdae290

Figure_S2_Flowchart_ABCD_cohort_23-10-2024_fdae290

## Notes

**Hanan Bozhar,** MSc (PhD Candidate)

**Susanne R. de Rooij,** PhD (Associate Professor)

**Anja Lok,** MD, PhD (Psychiatrist & Sr Researcher)

**Tanja Vrijkotte,** PhD (Associate Professor)

**Helle Larsen,** PhD (Associate Professor)
