# Supplementary figures and images for: On the edge of the social media landscape: associations with adolescent substance use and moderation by parental rules
Source: J Public Health (Oxf). 2024 Nov 19;47(1):90–8. doi: 10.1093/pubmed/fdae290 (PMC11879007; doi:10.1093/pubmed/fdae290)

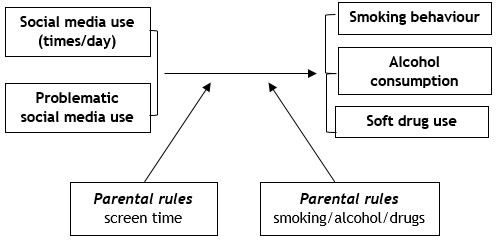

Supplement: Figure_S1_Research_model_23-10-2024_fdae290 [file figure_s1_research_model_23-10-2024_fdae290.jpeg]

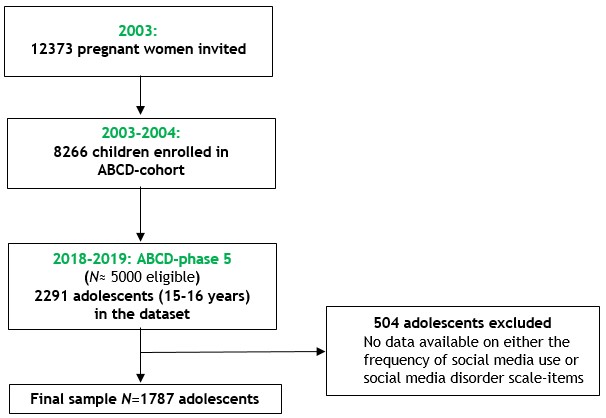

Supplement: Figure_S2_Flowchart_ABCD_cohort_23-10-2024_fdae290 [file figure_s2_flowchart_abcd_cohort_23-10-2024_fdae290.jpeg]
